# MicroRNA-4723 Inhibits Prostate Cancer Growth through Inactivation of the Abelson Family of Nonreceptor Protein Tyrosine Kinases

**DOI:** 10.1371/journal.pone.0078023

**Published:** 2013-11-01

**Authors:** Sumit Arora, Sharanjot Saini, Shinichiro Fukuhara, Shahana Majid, Varahram Shahryari, Soichiro Yamamura, Takeshi Chiyomaru, Guoren Deng, Yuichiro Tanaka, Rajvir Dahiya

**Affiliations:** Department of Urology, Veterans Affairs Medical Center, San Francisco and University of California San Francisco, San Francisco, California, United States of America; H.Lee Moffitt Cancer Center & Research Institute, United States of America

## Abstract

The Abelson (c-Abl) proto-oncogene encodes a highly conserved nonreceptor protein tyrosine kinase that plays a role in cell proliferation, differentiation, apoptosis and cell adhesion. c-Abl represents a specific anti-cancer target in prostate cancer as aberrant activity of this kinase has been implicated in the stimulation of prostate cancer growth and progression. However, the mechanism of regulation of c-Abl is not known. Here we report that Abl kinases are regulated by a novel microRNA, miR-4723, in prostate cancer. Expression profiling of miR-4723 expression in a cohort of prostate cancer clinical specimens showed that miR-4723 expression is widely attenuated in prostate cancer. Low miR-4723 expression was significantly correlated with poor survival outcome and our analyses suggest that miR-4723 has significant potential as a disease biomarker for diagnosis and prognosis in prostate cancer. To evaluate the functional significance of decreased miR-4723 expression in prostate cancer, miR-4723 was overexpressed in prostate cancer cell lines followed by functional assays. miR-4723 overexpression led to significant decreases in cell growth, clonability, invasion and migration. Importantly, miR-4723 expression led to dramatic induction of apoptosis in prostate cancer cell lines suggesting that miR-4723 is a pro-apoptotic miRNA regulating prostate carcinogenesis. Analysis of putative miR-4723 targets showed that miR-4723 targets integrin alpha 3 and Methyl CpG binding protein in addition to Abl1 and Abl2 kinases. Further, we found that the expression of Abl kinase is inversely correlated with miR-4723 expression in prostate cancer clinical specimens. Also, Abl1 knockdown partially phenocopies miR-4723 reexpression in prostate cancer cells suggesting that Abl is a functionally relevant target of miR-4723 in prostate cancer. In conclusion, we have identified a novel microRNA that mediates regulation of Abl kinases in prostate cancer. This study suggests that miR-4723 may be an attractive target for therapeutic intervention in prostate cancer.

## Introduction

Prostate cancer (PCa) is the most common male malignancy and the second leading cause of cancer death among men in the United States. Prostate cancer bone metastasis is the major cause of mortality in afflicted patients [1]. Despite many advances, clinical management of prostate cancer is challenging and is a leading cause of cancer-related morbidity and mortality. Major challenges include limited therapeutic options for metastatic, castration-resistant disease and the inability of current diagnostic tests to readily distinguish indolent from aggressive tumors [2]. Therefore, there has been much interest in the identification of novel, alternate prostate cancer biomarkers which could lead to development of better prognostic, diagnostic and therapeutic interventions for the disease.

The Abelson (Abl) family of highly conserved nonreceptor protein tyrosine kinases play important roles in various biological processes including cell survival, proliferation, cell adhesion and motility [3]. Mammalian cells have two Abl family kinases- Abl1 (c-Abl) and Abl2 (Abl-Related-Gene, Arg)- that are ubiquitously expressed [3], [4]. Central to the biochemical and physiological functions of Abl kinases are their combination of a regulated SH3-SH2-TK (Src homology 3–Src homology 2–tyrosine kinase) domain cassette with cytoskeletal protein– and DNA-binding domains, a combination that confers unique signaling capabilities to these proteins [5]. The activity of Abl kinases is tightly regulated by a complex set of intramolecular interactions that impinge on the Abl kinase domain and lead to effective auto-inhibition of tyrosine kinase activity [6]. Disruption of autoinhibition, such as by translocation of *Abl1* or *Abl2* next to a variety of different genes (e.g. *Bcr*, *Tel*, *ETV6*), leads to constitutive activation, which drives malignancies, particularly leukemias [4], [7], [8]. Mutations in the *Abl1* gene are commonly associated with chronic myelogenous leukemia (CML) where the *Abl1* gene is activated by being translocated within the *Bcr* (breakpoint cluster region) gene on chromosome 22, creating a new fusion gene, *Bcr-Abl*, that encodes an unregulated, tyrosine kinase.

Although Abl1 and Abl2 are well known for driving leukemia development, their role in solid tumors has been appreciated only recently [4]. c-Abl and/or Abl2 are activated in some solid tumor cell lines via unique mechanisms that do not involve gene mutation/translocation, and their activation promotes proliferation, matrix degradation, invasion, tumorigenesis, and/or metastasis, depending on the tumor type [4]. Accumulating evidence suggests that activation of c-Abl/Abl2 promotes prostate cancer progression [4]. c-Abl and/or Abl2 expression was significantly increased (assessed by immunohistochemistry [IHC]) in various tumors including prostate cancer [4], [9], [10]. Significantly, silencing c-Abl inhibited osteoblast proliferation and promoted osteoblast differentiation, indicating that c-Abl inhibition may prevent bone metastatic growth [11]. Dasatinib (BMS-354825), a dual Src family kinase (SFK) [12] and Abl kinase inhibitor [13] is being clinically tested for the treatment of prostate cancer bone metastasis and is currently in Phase 3 clinical trials [14]–[16]. Another SFK/Abl inhibitor bosutinib (SKI-606) blocked migration, invasion, anchorage-independent growth, and proliferation of PC3 and DU-145 prostate cancer cells accompanied by significant decrease in the phosphorylation of signaling molecules (AKT, mitogen-activated protein kinase, focal adhesion kinase) [17]. Furthermore, bosutinib inhibited *in vivo* PC3 tumor growth by subcuteaneous or intraskeletal injection [4], [17]. Activation of c-Abl by platelet derived growth factor (PDGF) promoted prostate cancer cell survival [18]. Abl kinases also play a role in regulating prostate cancer motility/invasion and progression [19]–[21]. Thus, Abl kinases play important roles in prostate cancer and represent important targets for specific anti-cancer therapy. However, the molecular mechanism of over-expression of Abl kinases in prostate cancer is not known. Here we report that Abl kinases are regulated by a novel microRNA, miR-4723, in prostate cancer.

MicroRNAs (miRNAs) constitute an evolutionarily conserved class of small noncoding RNAs that negatively regulate expression of multiple genes via sequence-specific interactions with the 3′- untranslated regions (UTRs) of cognate mRNA targets [22]. It has been firmly established that miRNAs control various key cellular processes such as proliferation, apoptosis, differentiation, development [23], and are implicated in human diseases, including cancer [24]. miRNAs have been identified that function as classical oncogenes or tumor suppressor genes [24]. Accumulating evidence suggests that there are correlations between miRNA expression and clinical recurrence, development of metastases and/or survival [25], [26]. Due to their tissue and disease-specific expression patterns and regulatory potential, miRNAs are being assessed as potential biomarkers for diagnosis and prognosis of human malignancies [27].

miR-4723 is a recently discovered miRNA [28] which has not been studied. We analyzed expression of miR-4723-5p (major form of miR-4723, referred to as miR-4723) expression in a cohort of prostate cancer clinical specimens and found that miR-4723 expression is widely attenuated in prostate cancer and is significantly correlated with poor survival outcome and tumor progression. Functional studies using prostate cancer cell lines showed that reconstitution of miR-4723 expression led to significant decreases in cell growth, clonability, invasion and migration. Significantly, miR-4723 reexpression led to dramatic induction of apoptosis in prostate cancer cell lines suggesting that miR-4723 is a pro-apoptotic miRNA that regulates prostate carcinogenesis. Further, our data suggests that this novel miRNA is an important regulator of Abl1 and Abl2 kinases that in turn regulates prostate carcinogenesis. To our knowledge this is the first report that (i) defines novel miRNA mediated regulation of Abl kinases in prostate cancer and (ii) identifies a novel tumor suppressor miRNA in prostate cancer. This regulatory mechanism will potentially be useful for therapeutic intervention in prostate cancer.

## Results

### miR-4723 Expression is Widely Attenuated in Prostate Cancer

**Figure 1:**
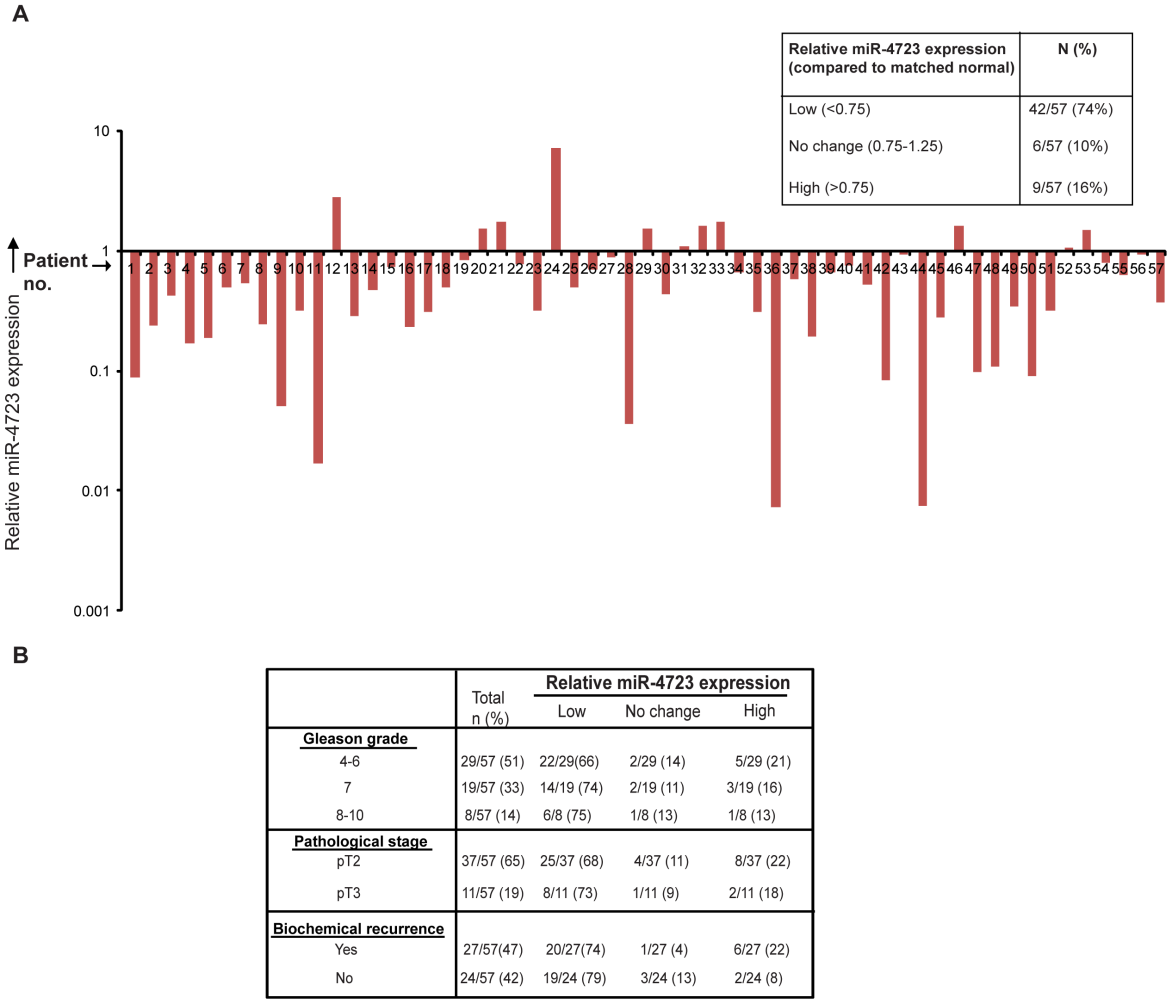
miR-4723 expression is widely attenuated in prostate cancer. (A) Quantitative RT-PCR analysis of relative miR-4723 expression levels in laser capture microdissected (LCM) PCa tissues (n = 57) and patient matched adjacent normal regions. Data were normalized to RNU48 control. Table summarizes the relative miR-4723 expression levels in these specimens. (B) Correlation of miR-4723 expression with clinicopathological characteristics of prostate cancer patients including Gleason grade, pathological stage and biochemical recurrence.

To evaluate the role of miR-4723 in prostate cancer, miR-4723 expression was assayed in human human clinical prostate samples (Fig. 1A). Clinicopathological characteristics of the patients are summarized in Table S1. We examined the expression levels of miR-4723 in laser capture microdissected (LCM) prostate cancer tissues (n = 57) and matched adjacent normal regions by real-time PCR. While the expression of miR-4723 was unaltered in 6/57 cases (10%) and higher in 9/57 cases (16%), a major fraction of tissue samples (42/57, ∼74%) showed lower miR-4723 levels relative to matched normal tissues. The differences were statistically significant with the Wilcoxon Signed Rank test (p<0.0001). This suggests that miR-4723 is widely downregulated in prostate cancer and is a potential prostate cancer tumor suppressor.

### Downregulation of miR-4723 Expression is Associated with Tumor Progression in Prostate Cancer

We determined if miR-4723 expression in clinical tissues was correlated with clinicopathological characteristics such as Gleason grade, pathological stage and biochemical recurrence of the carcinoma (Fig. 1B). Decreased miR-4723 expression was observed in 66% of cases of low Gleason grade (4–6), 74% of cases of Gleason grade 7 and in 75% of cases of higher Gleason grade (8–10). This trend suggests that expression of miR-4723 tends to decrease in higher grade prostate cancer. Similarly, decreased miR-4723 expression was observed in 68% of cases of pathological stage pT2 vs 73% of cases of pT3 while there was no significant correlation with biochemical recurrence.

### miR-4723 is a Potential Diagnostic and Prognostic Marker in Prostate Cancer

**Figure 2:**
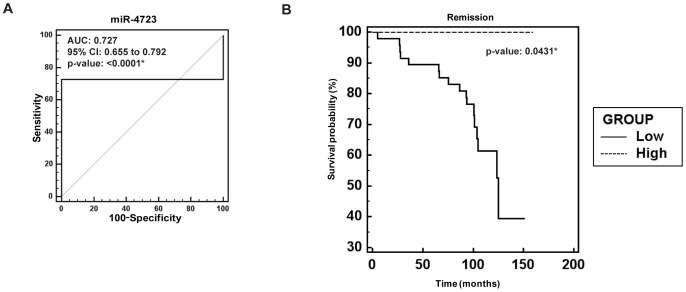
Clinical significance of miR-4723 expression in prostate cancer. (A) ROC curve analysis showing the ability of miR-4723 expression to discriminate between malignant and non-malignant prostate tissue samples. (B) Kaplan-Meier survival curves for prostate cancer patients, stratified based on miR-4723 expression (low and high). p value based on log rank test (*p<.05).

In view of the observed widespread downregulation of miR-4723 in prostate cancer clinical specimens, we evaluated the potential clinical significance of miR-4723 expression. To determine the potential capability of miR-4723 as a diagnostic biomarker for prostate cancer, we performed ROC (Receiver Operating Characteristic) analyses on the cohort of clinical samples (Fig. 2A). ROC analyses showed that miR-4723 expression can be a single significant parameter to discriminate between normal and tumor tissues with an area under the ROC curve (AUC) of 0.727 (95% CI: 0.655–0.792, p<0.0001). Further, we stratified our prostate cancer clinical cohort based on miR-4723 expression and performed Kaplan-Meier survival analysis. This analysis showed that survival was significantly reduced in patients with low miR-4723 expression (Fig. 2B) (p = 0.0431). These analyses suggest that miR-4723 has significant potential to be used as a diagnostic and prognostic marker for prostate cancer.

### miR-4723 Overexpression Suppresses Tumorigenicity of Prostate Cancer Cells

**Figure 3:**
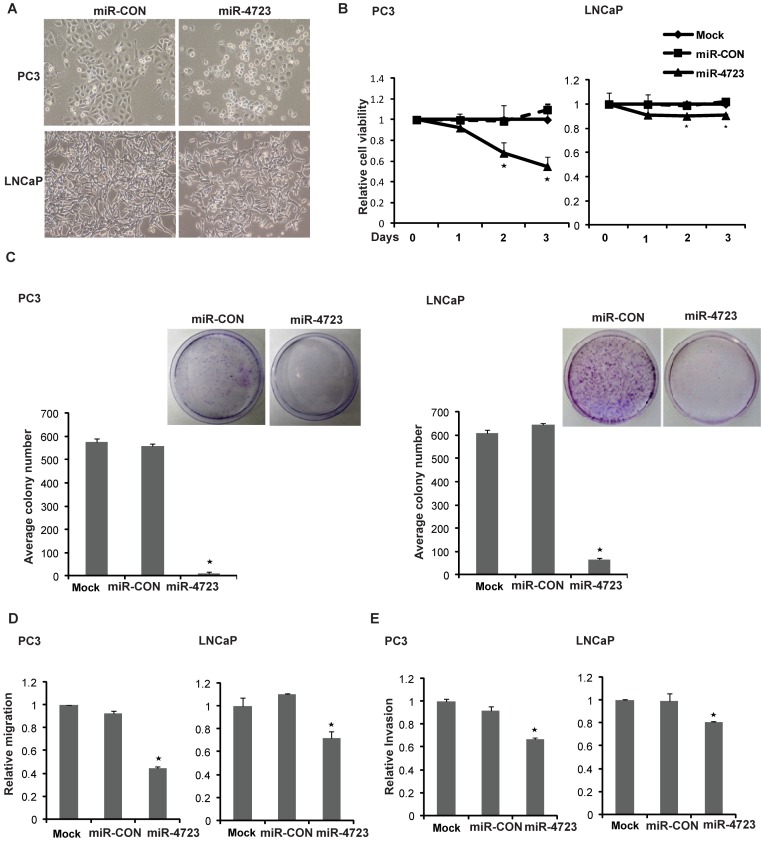
Overexpression of miR-4723 expression suppresses tumorigenicity of prostate cancer cells. To evaluate the functional significance of mir-4723 in prostate cancer, miR-4723 precursor was overexpressed in PC3/LNCaP cell lines by transient transfection followed by functional assays (performed 72 hrs post-transfection). (A) Morphological alterations in PC3/LNCaP cells upon miR-4723 expression assessed by phase-contrast microsopy. (B) Cellular viability assay, (C) Colony formation assay, (D) Transwell-migration assay and (E) Invasion assay in PC3/LNCaP cells mock transfected or transfected with miR-CON or miR-4723 (*P<.05).

To assess the potential for a tumor suppressive role of miR-4723, we overexpressed miR-4723 in prostate cancer cell lines (PC3 and LNCaP) followed by functional assays (Fig. 3). Transient transfection of miR-4723 precursor led to overexpression of miR-4723 as determined by real-time PCR (Fig. S1). Expression of miR-4723 led to marked morphological changes in both cell lines (Fig. 3A). Specifically, a pronounced decrease in the fraction of elongated, spindle shaped cells was paralleled by an increase in rounded, apoptotic cells. The morphological alterations observed with miR-4723 overexpression suggested an increase in apoptotic cells. A significant decrease in cell viability was observed over time in PC3/LNCaP cells overexpressing miR-4723 (Fig. 3B) as compared to cells expressing control miR (miR-CON). miR-4723 overexpression also decreased clonogenicity of PC3/LNCaP cells compared to miR-CON (Fig. 3C). Transwell migration and invasion assays showed that miR-4723 expression decreased the migration (Fig. 3D) and invasion (Fig. 3E) of both cell lines. These observations suggest that miR-4723 overexpression suppresses the tumorigenicity of prostate cancer cells.

### Overexpression of miR-4723 Induces Apoptosis in Prostate Cancer Cells

**Figure 4:**
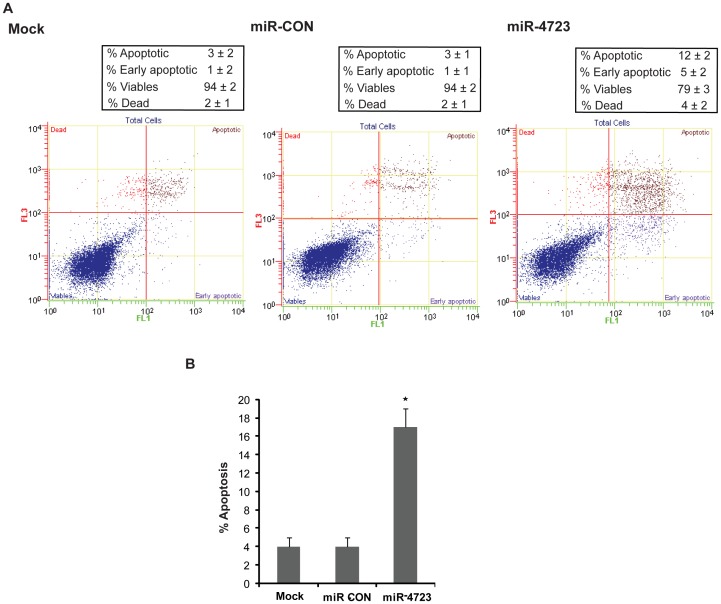
miR-4723 reexpression induces apoptosis in prostate cancer cells. (A) Apoptosis assay in PC3 cells after miR-CON (left panels) or miR-4723 (right panels) transfection for 72 hrs as assessed by ANNEXIN V-FITC/7-AAD staining. (B) Representation of average apoptotic fractions (early+late apoptotic) in each group (*p<.05).

We measured apoptosis in control (mock or miR-CON transfected) and miR-4723-tranfected cells by flow cytometric analysis of Annexin-V-FITC-7-AAD stained PC3 cells (Fig. 4A–B). It was observed that the average apoptotic cell fractions (Early apoptotic+Apoptotic) were significantly increased upon miR-4723 reexpression (12%+5%) compared to miR-CON or mock transfected cells (3%+1%) with a concomitant decrease in the viable cell population. This points to a pro-apoptotic role of miR-4723 and suggests that miR-4723 affects apoptotic pathways in regulating tumorigenicity.

### miR-4723 Induces G2-M Arrest in Prostate Cancer Cells

**Figure 5:**
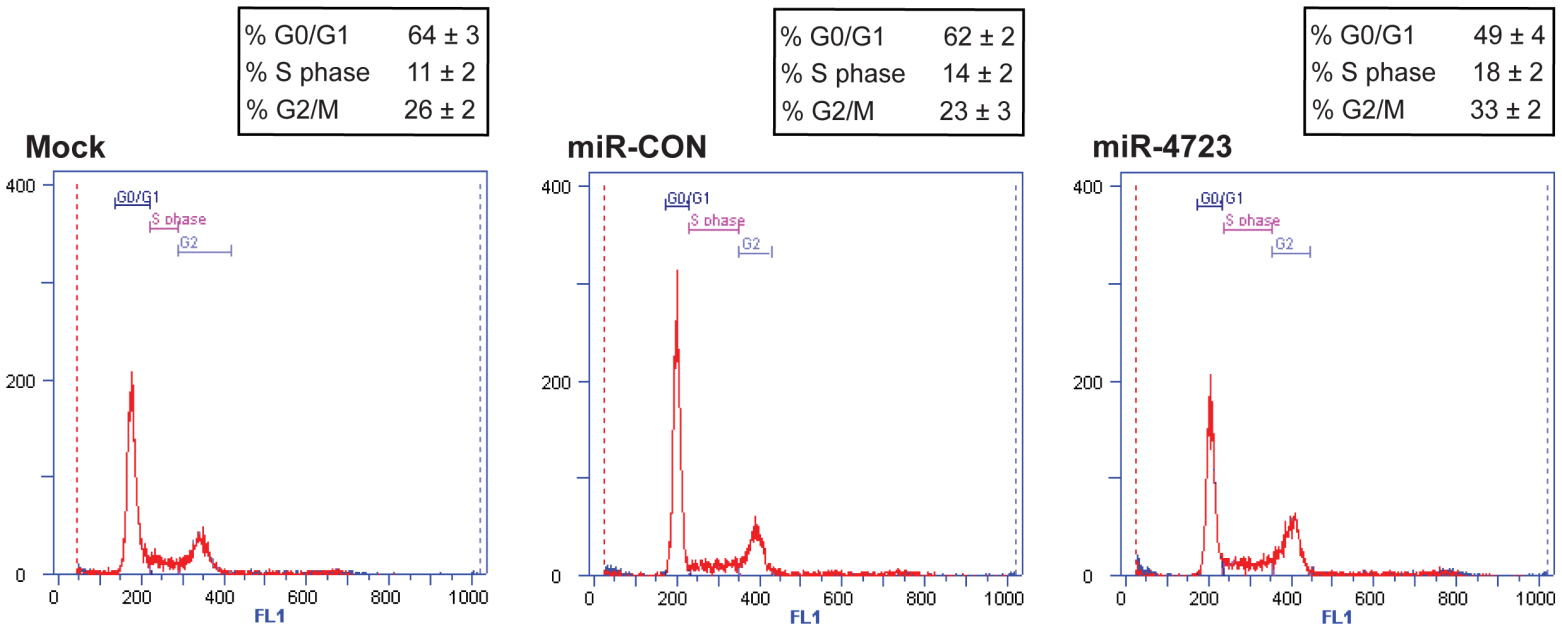
miR-4723 overexpression induces G2-M phase arrest in prostate cancer cells. Cell cycle analysis after mock (left panel)/miR-CON (middle panel) or miR-4723 (right panel) treatments showing induction of G2/M phase arrest by miR-4723. 72 hours post-transfection. cells were stained with DAPI nuclear stain for FACS analysis.

FACS (fluorescence activated cell sorting) analysis showed that expression of miR-4723 in PC3 cells lead to a significant increase in the number of cells in the G2-M phase of the cell cycle compared to miR-CON (Fig. 5). This suggests that miR-4723 expression induces G2-M arrest in prostate cancer cells.

### miR-4723 Targets Abl Kinases in Prostate Cancer Cells

**Figure 6:**
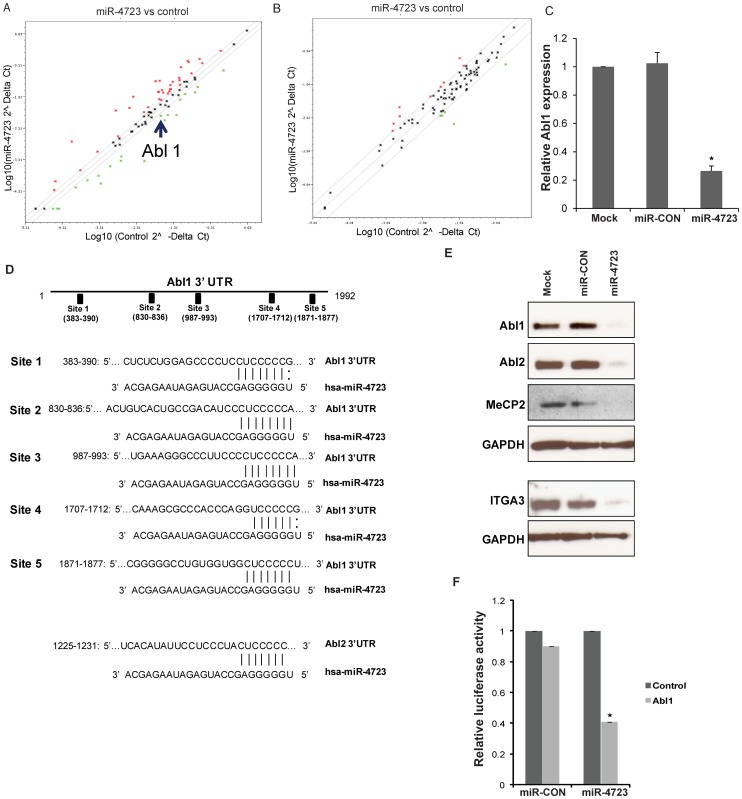
miR-4723 directly targets Abl kinases in prostate cancer cells. (A) Profiling of apoptotic genes and, (B) Cell cycle related genes after overexpression of miR-4723 in PC3 cells showing downregulation of Abl1 by miR-4723. (C) Relative mRNA expression of Abl1 gene as assessed by RT-PCR. (D) Schematic representation of the Abl1 and Abl2 3′-UTRs showing the relative positions of putative miR-4723 binding sites. (E) Immunoblots of endogenous Abl1, Abl2 and other miR-4723 targets in PC3 cells transfected as indicated. GAPDH was used a loading control. (F) Abl1 3′ UTR construct encompassing miR-4723 binding sites or the control construct was cotransfected into PC3 cells with miR-4723 or miR-CON and assayed for relative luciferase activity (*p<.05).

To identify effectors of miR-4723, a list of potential miRNA targets was created by combining predicted targets from the miRDB target prediction algorithm [29], [30] and profiling the expression of genes related to apoptosis and the cell cycle using pathway focussed PCR Arrays. Consistent with the observed effects of miR-4723 on apoptosis and the cell cycle, we found several key molecules of these cellular processes as potential miR-4723 targets. Profiling of apoptosis and cell cycle related genes consistently showed downregulation of the Abl1 gene (Fig. 6A–B). To validate these results, we performed RT-PCR analysis to examine the relative mRNA expression levels of Abl1 and found that miR-4723 overexpression leads to decreased Abl1 expression (Fig. 6C). This suggests that miR-4723 represses Abl1 via mRNA degradation.

Our *in silico* analyses showed that Abl1 possesses five potential miR-4723 target sites within its 3′-UTR (Fig. 6D). Sites 2–5 are evolutionarily conserved in primates (chimpanzee, gorilla, rhesus monkey) (Fig. S2). Also, we found that the closely related Abl2 possesses a potential miR-4723 target site within its 3′-UTR (Fig. 6D, lower panel). This site is conserved in chimpanzee and gorilla (Fig. S2).

Guided by these findings, we performed Western blot analysis for putative miR-4723 targets in PC3 cells that were either mock transfected or transfected with miR-4723/miR-CON (Fig. 6E). Interestingly, miR-4723 overexpression led to decreased protein levels of Abl1 and Abl2 that encode the Abelson family of nonreceptor tyrosine protein kinases. In addition, we found that miR-4723 represses Methyl CpG binding protein (MeCP2) and integrin, alpha 3 (ITGA3). The 3′UTR of MeCP2 possesses ten potential miR-4723 target sites while that of ITGA3 have four potential miR-4723 target sites (Fig. S3).

We investigated whether the c-Abl protooncogene is a direct functional target of miR-4723 in prostate cancer. Transient transfection of human PC3 cancer cells with the Abl1 3′UTR plasmid along with miR-4723 precursor led to a significant decrease in promoter activity when compared with the control vector (Fig. 6E) suggesting that miR-4723 directly represses this gene. These observations led us to conclude that miR-4723 regulates a cohort of genes which play an important role in cellular survival, adhesion, invasion and metastasis particularly Abl kinases.

### Abl1 Knockdown Partially Phenocopies miR-4723 Reexpression in Prostate Cancer Cells

**Figure 7:**
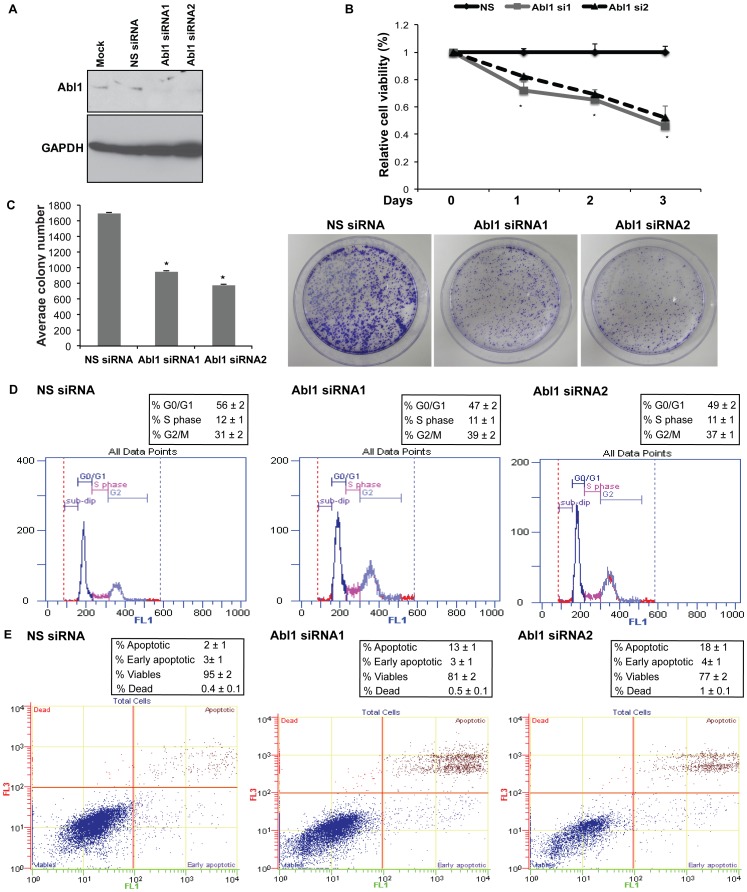
Abl1 knockdown partially phenocopies miR-4723 reexpression in prostate cancer cells. PC3 cells were transfected with Abl1 siRNA/nonspecific (NS) control siRNA for 72 h followed by various assays. Two sets of siRNA against Abl1- Abl1 siRNA1 and siRNA2- were used for functional assays. (A) Relative Abl1 protein expression after siRNA transfections as assessed by immunoblotting. GAPDH was used as a loading control. (B) Cell viability assay, (C) Colony formation assay, (D) Cell cycle analysis, (E) Apoptosis assay in PC3 cells after NS siRNA (left panel)/Abl1 siRNA1 (middle panel)/Abl1 siRNA2 (right panel) treatments.

To determine if Abl is a functionally relevant target of miR-4723 in prostate cancer, we inhibited Abl1 expression using siRNA to see if Abl1 knockdown functionally mimics the effects of miR-4723 overexpression. We treated PC3 cells with Abl1 siRNA followed by *in vitro* functional assays (Fig. 7). Two sets of siRNA against Abl1- Abl1 siRNA1 and siRNA2- were used to achieve efficient knockdown as assessed by immunoblot analysis (Fig. 7A). Non-specific siRNA (NS) was used as a control. A significant decrease in cell viability was observed in Abl1 siRNA treated PC3 cells as compared to cells treated with control siRNA (Fig. 7B). Abl1 knockdown also led to decreased clonogenicity of PC3 cells compared to control (Fig. 7C). Cell cycle analysis showed that Abl1 knockdown led to G2-M phase arrest similar to miR-4723 overexpression (Fig. 7D). Apoptosis assay showed that apoptotic cell fractions were significantly increased upon Abl1 knockdown compared to control siRNA-treated cells, an effect similar to that observed upon miR-4723 reintroduction in PC3 cells (Fig. 7E). These results suggest that Abl1 is an important determinant of the tumorigenic properties of prostate cancer cells and that Abl1 inhibition partially phenocopies the anti-tumorigenic effects of miR-4723 in prostate cancer.

### Expression of Abl Kinase is Inversely Correlated with miR-4723 Expression in Prostate Cancer

**Figure 8:**
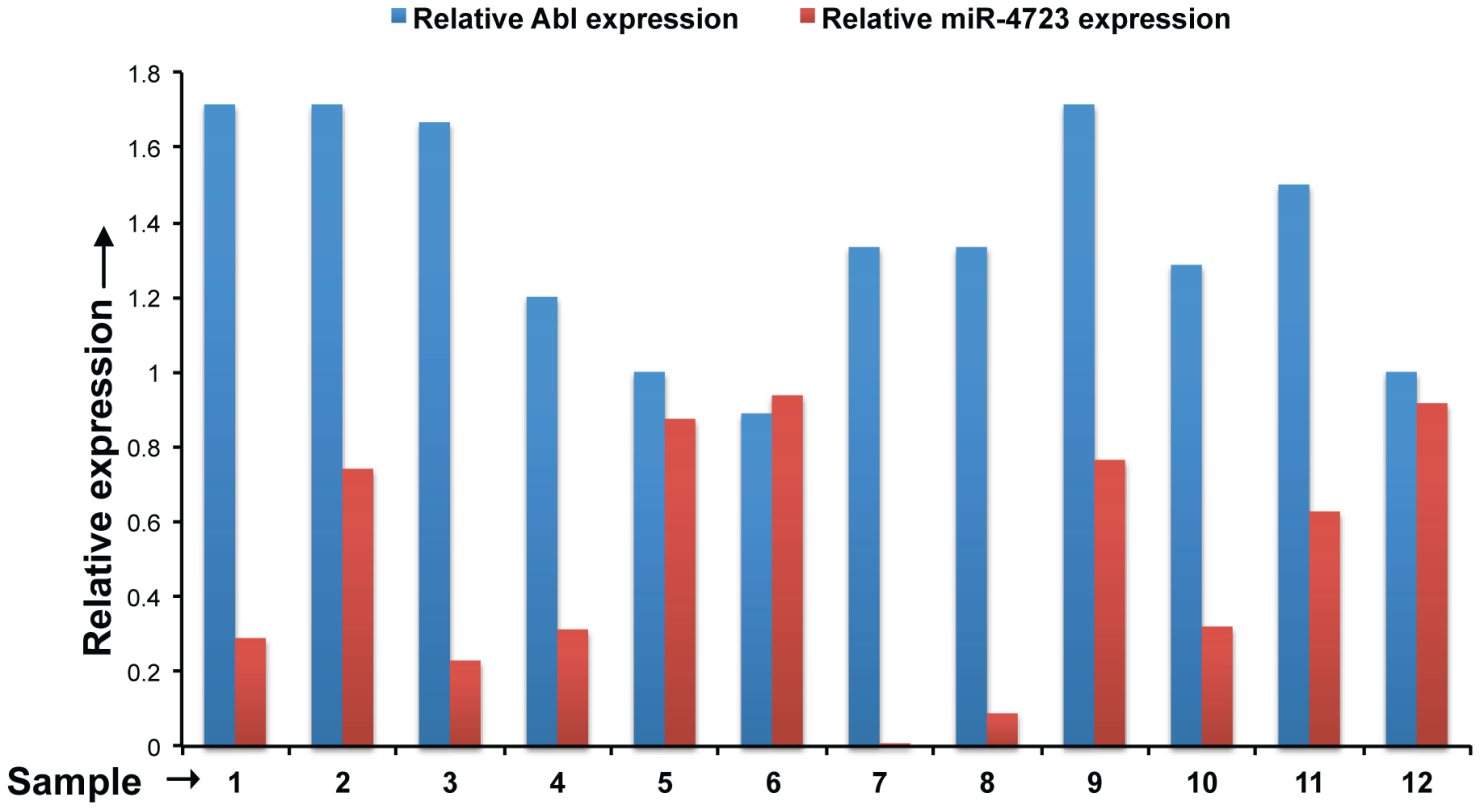
Expression of Abl kinase is inversely correlated with miR-4723 expression in prostate cancer. We examined the correlation between miR-4723 and Abl expression in a subset of our clinical cohort by performing immunohistochemical staining for Abl in PCa tissues (n = 12). Relative expression levels of Abl (as scored by IHC) and miR-4723 (as determined by RT-PCR) for the samples analysed are represented in the bar graph.

To further confirm Abl as a pathologically relevant target of miR-4723 *in vivo*, we examined the correlation between miR-4723 and Abl expression in a subset of our clinical cohort. We performed immunohistochemical staining for Abl in PCa tissues (n = 12) and observed a negative correlation between Abl and miR-4723 expression (Fig. 8). Clinical samples with low miR-4723 expression (relative to adjacent normal tissue) showed high levels of Abl expression (Fig. 8).

## Discussion

An expansive body of literature shows that miRNAs are significantly altered in prostate cancer [31]. As the molecular interactions of miRNAs with their cognate target genes are being explored, the present study was aimed to identify novel miRNAs that regulate prostate carcinogenesis. In this study, we identified a novel tumor-suppressive miRNA, miR-4723, in prostate cancer. miR-4723 is a recently discovered miRNA [28] which has not been explored yet. Expression profiling of miR-4723 expression in a cohort of prostate cancer clinical specimens showed that miR-4723 expression is widely downregulated in prostate cancer. In view of its low expression, we assessed the potential of miR-4723 as a prostate cancer biomarker. Our analyses suggests that low miR-4723 expression can be a significant parameter to discriminate between normal prostate and tumor tissues. Further, low miR-4723 expression was significantly correlated with poor survival outcome and tumor progression in clinical specimens. These findings suggest that this novel miRNA has significant potential as a disease biomarker for prostate cancer diagnosis and prognosis.

The observed downregulation of miR-4723 expression in PCa clinical samples also suggested that this novel miRNA may possess tumor-suppressive activity. To test this, we performed functional studies using prostate cancer cell lines PC3 and LNCaP. We overexpressed miR-4723 in these PCa cell lines followed by functional assays. Our data suggests that overexpression of miR-4723 in PCa cells suppresses tumorigenicity, confirming the tumor-suppressive role of miR-4723 in prostate cancer. miR-4723 overexpression led to significant decreases in cell growth, clonability, invasion and migration. Importantly, these studies indicate that miR-4723 is a pro-apoptotic miRNA that regulates prostate carcinogenesis. This is the first report implicating a tumor suppressor role for this miRNA in prostate cancer where we show that it has an important pro-apoptotic role.

In mammalian cells, miRNAs cause gene silencing of their target genes via both translational inhibition and mRNA degradation and an individual miRNA is capable of regulating dozens of distinct mRNAs [22]. To understand the functional role of miR-4723 in prostate carcinogenesis, we looked for the putative target genes that play an important role in cellular survival, adhesion, invasion and metastasis. Our data suggests that c-Abl protooncogene and Abl2/Arg are important functional targets of miR-4723 in prostate cancer. Abl1 and Abl2 encode the Abelson family of nonreceptor tyrosine protein kinases that are ubiquitously expressed and highly conserved in metazoan evolution [3]. These kinases play a role in regulating cell proliferation, differentiation, apoptosis, cell adhesion and stress responses [3]. Aberrant activity of these tyrosine kinases have been implicated in the stimulation of cancer growth and progression in various tumors, particularly leukemias. Studies suggest that activation of c-Abl/Abl2 promotes prostate cancer progression [4]. In prostate tumors, c-Abl and/or Abl2 expression was significantly increased as assessed by immunohistochemistry [IHC]) [4], [9], [10]. Activation of c-Abl by platelet derived growth factor (PDGF) promoted prostate cancer cell survival by inducing expression of the antiapoptotic protein, MCL-1, via a p68/β-catenin signaling pathway [18]. Abl kinases also play a role in motility/invasion of prostate cancer cells, as c-Abl/Arg–mediated phosphorylation of galectin-3 prevented its cleavage by PSA thereby increasing full-length extracellular galectin-3 and promoting migration and invasion of PCa cells [19], [21]. A role of c-Abl in prostate cancer progression has been demonstrated due to its ability to phosphorylate a Wiskott-Aldrich syndrome protein family, member 3 (WASF3), a protein that controls cellular motility and invasion and is upregulated in advanced metastatic PCa [20]. Thus, these kinases are important targets for specific anti-cancer therapy and Abl kinase inhibitors are currently in clinical trials for the treatment of prostate cancer bone metastasis. Dasatinib (BMS-354825), a dual Src family kinase (SFK) [12] and Abl kinase inhibitor [13] is currently in Phase 3 clinical trials [14]–[16]. Dasatinib suppresses proliferation and enhances differentiation of osteoblasts, indicating that c-Abl inhibition may prevent bone metastatic growth [11]. Bosutinib (SKI-606), another SFK/Abl inhibitor, inhibits the growth of prostate cancer cells *in vitro* and *in vivo* [17]. Overall, these studies suggest that Abl kinases play important roles in prostate cancer. However, the mechanisms of regulation of Abl kinase activities are not completely understood. Here we report a novel miRNA mediated regulation of these kinases in prostate cancer.

MicroRNA mediated regulation of Abl1 and Bcr-Abl has been explored in hematopoietic malignancies. Bueno *et al.* showed that Abl1 is a direct target of miR-203 in hematopoietic malignancies. miR-203 is silenced by genetic and epigenetic mechanisms in hematopoietic malignancies expressing either Abl1 or Bcr-Abl1, and restoration of miR-203 expression reduced Abl1 and Bcr-Abl1 levels and inhibited cell proliferation [32], [33]. In a recent study, miR-451 has also been shown to negatively regulate Bcr-Abl expression in chronic myeloid leukemia [34], [35]. However, such studies are lacking in prostate cancer. To our knowledge, this is the first report to show novel miRNA mediated inactivation of Abl kinases in prostate cancer. In view of our present results, we suggest that the observed effects of miR-4723 on apoptosis, proliferation, invasion and migration are mediated by its ability to repress Abl kinases.

An individual miRNA is capable of regulating several distinct mRNAs [36], [37], and it has been demonstrated that pleiotropic suppression of multiple downstream targets may explain the phenotypic changes observed by altered levels of certain miRNAs [38]–[42]. We found that miR-4723 also represses Methyl CpG binding protein (MeCP2) and integrin, alpha 3 (ITGA3). MeCP2 is a nuclear multifunctional protein involved in several cellular processes, such as chromatin reorganization, architecture, and transcriptional regulation [43]. In recent years, a role for MeCP2 has emerged in cell growth and proliferation [44]. In prostate cancer, studies suggest that MeCP2 is required for prostate cancer cell growth [45], [46]. Inhibition of MeCP2 expression by stable short hairpin RNA stopped the growth of both normal and cancer human prostate cells while ectopic MeCP2 expression conferred a growth advantage to human prostate cancer cells. Importantly, its expression allowed androgen-dependent cells to grow independently of androgen stimulation and to retain tumorigenic properties in androgen-depleted conditions [45]. In another study, silencing of MeCP2 in PC3 cells led to decreased proliferation and increased apoptosis [46]. Integrin A3 (ITGA3), a cell adhesion molecule of the integrin family, plays a number of essential roles in processes required for cancer progression, including proliferation, survival, invasion, and metastasis [47]. This integrin receptor forms complexes with various membrane, cytoskeletal and signaling molecules to regulate cell adhesion, migration, signal transduction across cell membranes, and cytoskeletal organization [47], [48]. Integrins are attractive targets for anti-cancer therapeutics and there have been preclinical and clinical development of antagonists targeting integrins [49].

In conclusion, our study identifies miR-4723 as an important miRNA that regulates prostate cancer growth through inactivation of Abl kinases, MeCP2, ITGA3. Our study establishes that low expression of miR-4723 is associated with poor survival and progression of prostate cancer. In view of the widespread attenuation of miR-4723 expression observed in our patient cohort and its anti-tumorigenic effects on PCa cells, we suggest that restoration of miR-4723 expression may be a novel therapeutic modality for PCa. In addition, our study has identified a new miRNA based mechanism regulating Abl1 and Abl2 expression in prostate cancer. These findings may be of particular importance in the development of prostate cancer therapies utilizing kinase inhibitors.

## Materials and Methods

### Cell Lines and Cell Culture

Prostate carcinoma cell lines (LNCaP, PC3) were obtained from the American Type Culture Collection (ATCC) and were maintained in RPMI 1640 media supplemented with 10% fetal bovine serum (FBS) (Atlanta biologicals) and 1% penicillin/streptomycin (UCSF cell culture facility). These human-derived cell lines were authenticated by DNA short-tandem repeat analysis by ATCC. The experiments with cell lines were performed within 6 months of their procurement/resuscitation. Cell lines were maintained in an incubator with a humidified atmosphere of 95% air and 5% CO2 at 37°C.

### Tissue Samples and Ethics Statement

Formalin-fixed, paraffin-embedded (FFPE) prostate cancer samples were obtained from the SFVAMC. Written informed consent was obtained from all patients and the study was approved by the UCSF Committee on Human Research (Approval number: H9058-35751-01). All slides were reviewed by a board certified pathologist for the identification of prostate cancer foci as well as adjacent normal glandular epithelium.

### Laser Capture Microdissection (LCM)

Tissue slides were reviewed by a board certified pathologist for the identification of prostate cancer foci as well as adjacent normal glandular epithelium. Microdissection was performed using the AutoPix System (Arcturus). 8 µm sections were placed on glass slides, deparaffinized, stained with hematoxylin, dehydrated, and placed in the AutoPix instrument for microdissection. Areas of interest were captured with infrared laser pulses onto CapSure Macro LCM Caps.

### RNA and miRNA Extraction

Total RNA was extracted from microdissected FFPE tissues using a miRNeasy FFPE Kit (Qiagen) and an miRNeasy mini kit (Qiagen) was used for miRNA extraction from cultured cells following the manufacturer’s instructions.

### Quantitative Real-time PCR

Mature miRNAs were assayed using the TaqMan MicroRNA Assays and Gene Expression Assays, respectively, in accordance with the manufacturer’s instructions (Applied Biosystems). Samples were normalized to RNU48 (Applied Biosystems) as indicated. Taqman miRNA assays used for expression analysis were hsa-miR-4723 (assay ID 463538_mat), RNU48 (assay ID 001006). The comparative Ct method was used to calculate the relative changes in gene expression with the 7500 Fast Real Time PCR System.

For real-time PCR based expression profiling of a set of genes of a particular pathway, pathway-specific PCR arrays (cell cycle, apoptosis) (Qiagen) were used as per the manufacturer’s instructions. Briefly, 1 ug of each RNA was reverse transcribed using the Reaction Ready First Strand cDNA synthesis kit (Qiagen) followed by PCR amplification using pre-dispensed gene-specific primers and QPCR master mix containing SYBR Green and reference dyes. Thermal cycling was performed on an ABI Prism 7500 Fast thermal cycler using the following conditions: 95°C for 10 minutes; 40 cycles of 95°C for 15 seconds, 60°C for 1 minute. The fold changes in gene expression between the experimental samples were calculated by the ddCt method.

### miRNA/siRNA Transfections

Cells were plated in growth medium without antibiotics ∼24 hrs before transfections. Transient transfections of miRNA precursors (Ambion) )/siRNA (Origene) were carried out by using Lipofectamine 2000 (Invitrogen) according to the manufacturers’s protocol. All miRNA/siRNA transfections were for 72 h. miR-4723 precursor (AM17100) or negative control (miR-CON) (AM17110) (Ambion) was used for miRNA transfections. Abl1 siRNA (SR300017) or universal scrambled negative control (NS siRNA, SR30004) were purchased from Origene.

### Cell Viability Assays

Cell viability was determined at 24, 48, 72 hours by using the CellTiter 96 AQueousOne Solution Cell Proliferation Assay Kit (Promega), according to the manufacturer’s protocol.

### Migration, Invasion and Clonogenicity Assays

Cytoselect Cell migration and invasion assay kit (Cell Biolabs, Inc.) was used for migration and invasion assays, according to the manufacturer’s protocol. Briefly, 48 hrs post-transfection, cells were counted and placed on control inserts or Matrigel inserts at 1×10^5^ cells/ml in serum-free medium and were allowed to migrate for 20 h at 37°C. Cells were removed from the top of the inserts and cells that migrated/invaded though the polycarbonate/basement membrane were fixed, stained and quantified at OD 560 nm after extraction. For clonogenicity assay, cells were counted, seeded at low density (1000 cells/plate) and allowed to grow until visible colonies appeared. Then cells were stained with Giemsa and colonies were counted.

### Flow Cytometry

Fluorescence-activated cell-sorting (FACS) analysis was done 72 hours post-transfection. The cells were harvested, washed with cold PBS, and resuspended in DAPI nuclear stain for cell cycle analysis. Cells were stained with 7-AAD and Annexin-V-FITC using ANNEXIN V-FITC/7-AAD KIT (Beckman Coulter) for apoptosis analysis according to the manufacturer’s protocol. Stained cells were immediately analyzed by FACS (Cell Lab Quanta SC; Beckman Coulter, Inc).

### Western Blotting

Whole cell extracts were prepared in RIPA buffer [50 mmol/L Tris (pH 8.0), 150 mmol/L NaCl, 0.5% deoxycholate, 0.1% SDS, and 1.0% NP-40] containing protease inhibitor cocktail (Roche). Total protein was electrophoresed by SDS-PAGE and Western blotting was carried out according to standard protocols. The following antibodies were used for Western blotting: Abl1 (Cell Signaling, cat no. 2862), Abl2 (Cell Signaling, cat no. 7729), ITGA3 (Millipore, cat no. AB1920), MeCP2 (Cell Signaling, cat no. 3456), GAPDH (Santa Cruz Biotechnology, sc-32233).

### Luciferase Assays

For Abl1, the corresponding 3′UTR reporter construct (catalog no. HmiT006955) was obtained from Genecopoeia along with control construct (CmiT000001-MT01). All these target clones were in the SV-40 promoter based vector pEZX-MT01 (Genecopoeia). Control constructs and various 3′-UTR reporter constructs (0.2 ug) were cotransfected into PC3 cells cultured in 24-well plates along with 50 nM miR-4723 or miR-CON (Ambion) using Lipofectamine 2000 (Invitrogen). 48 hr post-transfection, firefly and renilla luciferase activities were measured by using the dual luciferase reporter assay system (Promega) according to the manufacturer’s protocol. Firefly luciferase was normalized to renilla luciferase activity.

### Immunohistochemistry

Immunostaining was done on formalin-fixed, paraffin-embedded prostate cancer tissues. The slides were deparrafinized and antigen retrieval was carried out by microwaving the slides in 10 mmol/L sodium citrate buffer. Slides were incubated overnight with anti-Abl antibody (Cell Signaling, cat no. 2862). The staining was done using the ImmunoCruz Staining System (Santa Cruz Biotechnology, Santa Cruz, CA) as per manufacturer’s instructions.

### Statistics

All quantified data represents an average of at least triplicate samples or as indicated. Data are represented as mean ± S.E.M. All statistical analyses were performed using StatView (version 5; SAS Institute Inc.) and MedCalc version 10.3.2. Two-tailed Student’s t-test was used for comparisons between groups. The Wilcoxon Signed Rank test was used to assess the difference between miRNA expression in tumor/normal adjacent tissue. Receiver operating curves (ROC) were calculated to determine the potential of miR-4723 to discriminate between malignant and non-malignant samples. Results were considered statistically significant at p≤0.05.

## Supporting Information

Figure S1 **miR-4723 overexpression in prostate cancer cell lines.** Relative miR-4723 expression in PC3 cells (left panel) or LNCaP cells (right panel) transfected with either control miR/miR-4723/mock transfected cells as assessed by real time PCR.
(TIF)

Figure S2 **Conservation of miR-4723 binding sites in Abl1 and Abl2 3′ UTR.** Schematic representation showing conservation of putative miR-4723 binding sites in Abl1 and Abl2 3′-UTRs. **A.** Abl1 possesses five potential miR-4723 target sites within its 3′-UTR (Fig. 6D). Sites 2–5 are evolutionarily conserved in primates (chimpanzee, gorilla, rhesus monkey). **B.** Abl2 possesses a potential miR-4723 target site within its 3′-UTR (Fig. 6D, lower panel). This site is conserved in chimpanzee and gorilla.
(TIF)

Figure S3 **MeCP2 and ITGA3 are miR-4723 targets.** Schematic representation showing putative miR-4723 binding sites in MeCP2 and ITGA3 3′-UTRs.
(TIF)

Table S1 **Clinicopathologic characteristics of prostate cancer patients.** Clinicopathological data for matched LCM-microdissected tissues used for real-time PCR analysis of miR-4723 expression in Fig. 1. **‘**Unknown’ refers to the information not available for some samples.
(DOC)
