# Supplementary figures and images for: MicroRNA-4723 Inhibits Prostate Cancer Growth through Inactivation of the Abelson Family of Nonreceptor Protein Tyrosine Kinases
Source: PLoS One. 2013 Nov 1;8(11):e78023. doi: 10.1371/journal.pone.0078023 (PMC3815229; doi:10.1371/journal.pone.0078023)

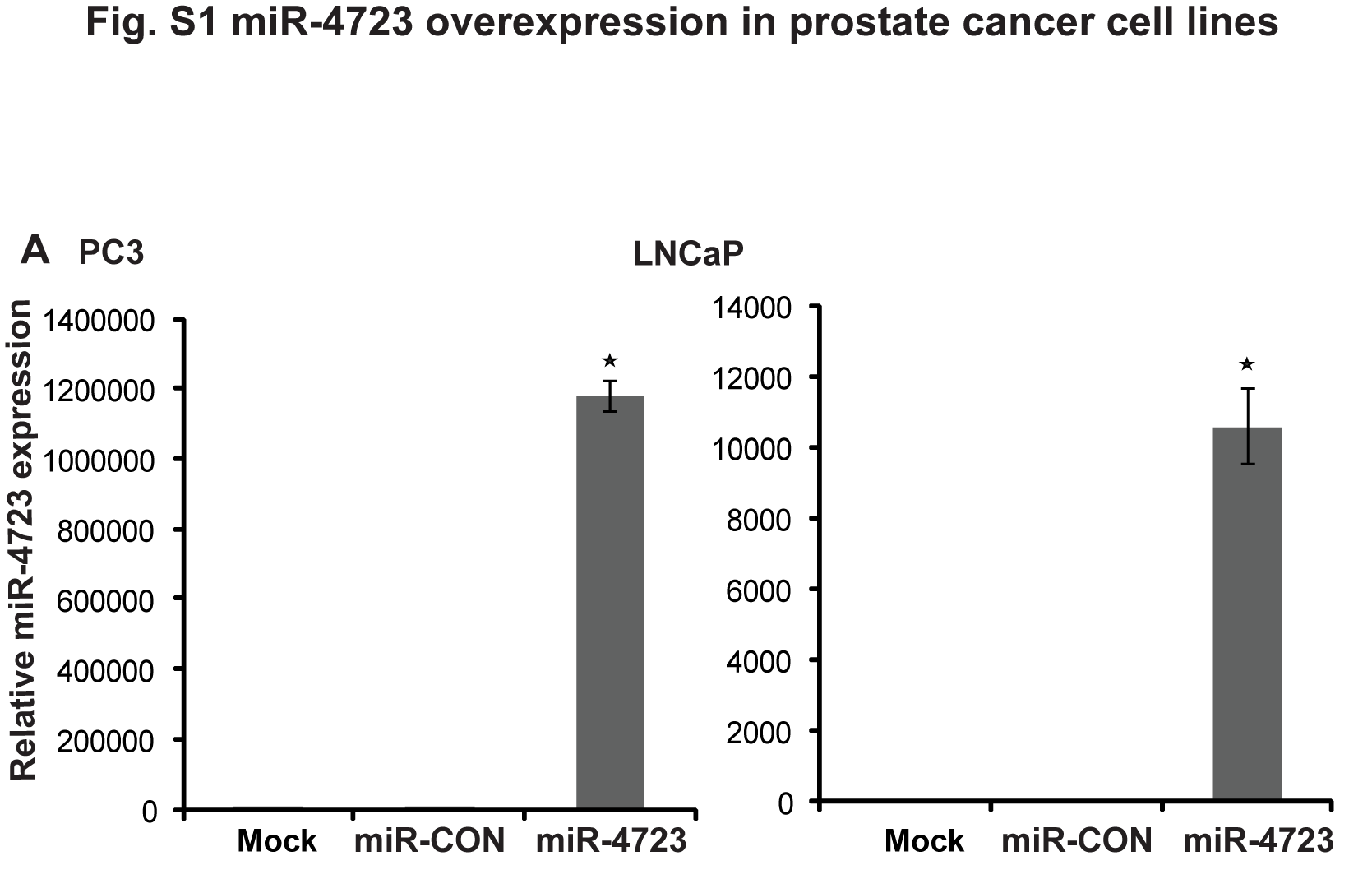

Supplement: Figure S1 — miR-4723 overexpression in prostate cancer cell lines. Relative miR-4723 expression in PC3 cells (left panel) or LNCaP cells (right panel) transfected with either control miR/miR-4723/mock transfected cells as assessed by real time PCR. (TIF) [file pone.0078023.s001.tif]

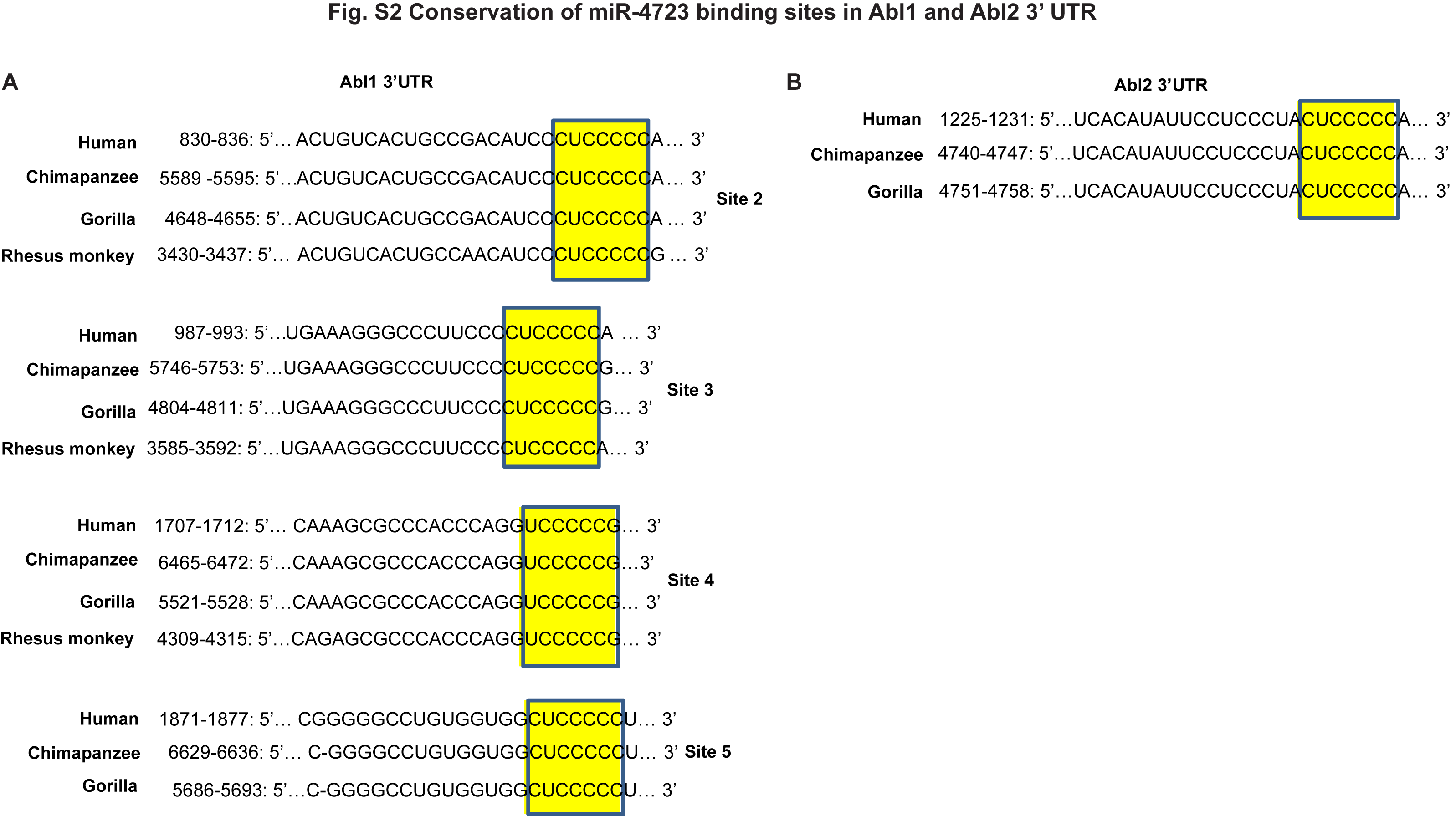

Supplement: Figure S2 — Conservation of miR-4723 binding sites in Abl1 and Abl2 3′ UTR. Schematic representation showing conservation of putative miR-4723 binding sites in Abl1 and Abl2 3′-UTRs. A. Abl1 possesses five potential miR-4723 target sites within its 3′-UTR (Fig. 6D). Sites 2–5 are evolutionarily conserved in primates (chimpanzee, gorilla, rhesus monkey). B. Abl2 possesses a potential miR-4723 target site within its 3′-UTR (Fig. 6D, lower panel). This site is conserved in chimpanzee and gorilla. (TIF) [file pone.0078023.s002.tif]

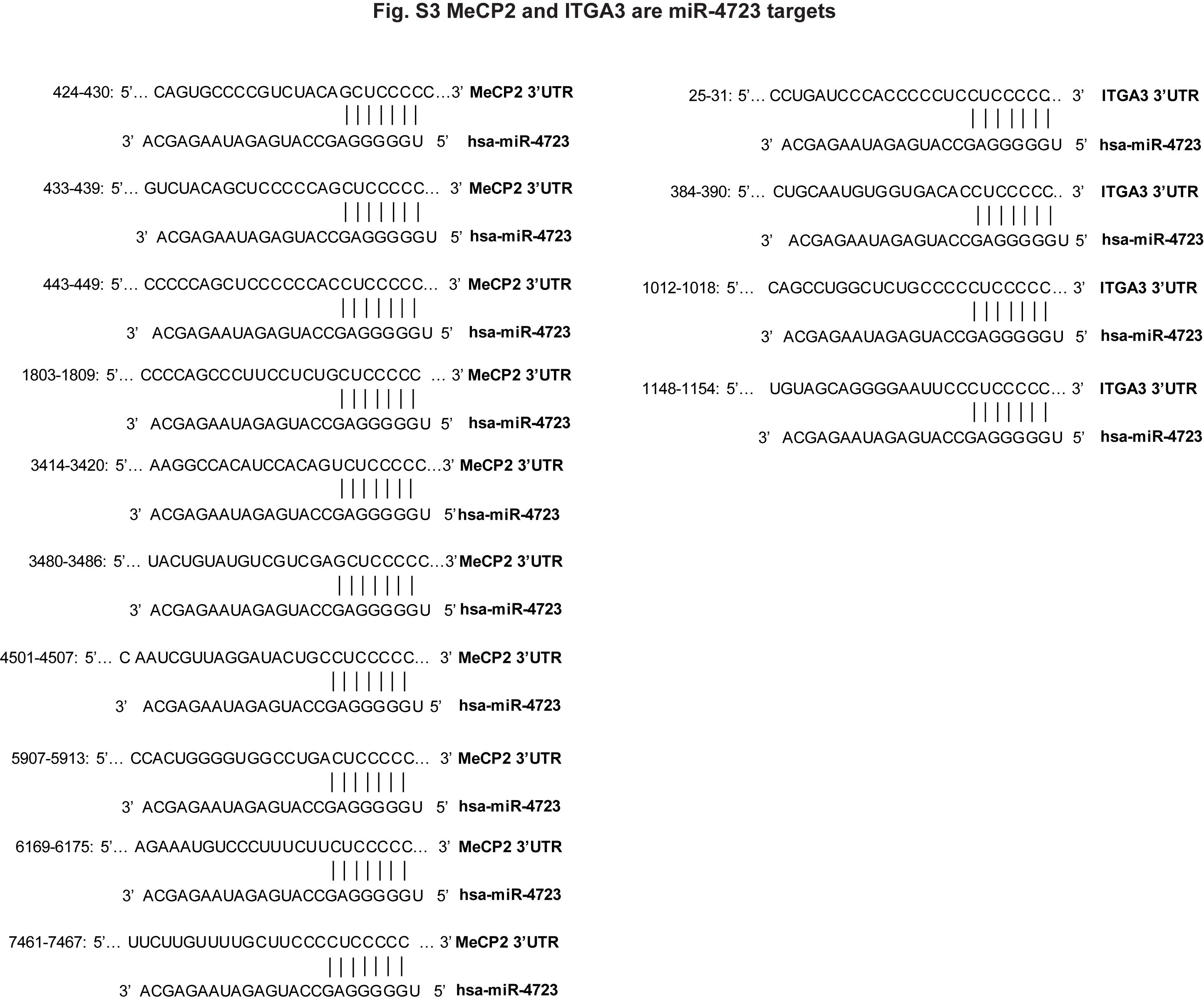

Supplement: Figure S3 — MeCP2 and ITGA3 are miR-4723 targets. Schematic representation showing putative miR-4723 binding sites in MeCP2 and ITGA3 3′-UTRs. (TIF) [file pone.0078023.s003.tif]
