# Supplementary material for: MicroRNA-4723 Inhibits Prostate Cancer Growth through Inactivation of the Abelson Family of Nonreceptor Protein Tyrosine Kinases
Source: PLoS One. 2013 Nov 1;8(11):e78023. doi: 10.1371/journal.pone.0078023 (PMC3815229; doi:10.1371/journal.pone.0078023)
Supplement: Table S1 — Clinicopathologic characteristics of prostate cancer patients. Clinicopathological data for matched LCM-microdissected tissues used for real-time PCR analysis of miR-4723 expression in Fig. 1. ‘Unknown’ refers to the information not available for some samples. (DOC) [file pone.0078023.s004.doc]

Clinicopathologic characteristics of prostate cancer patients (N=57).

**Characteristics Number of patients**

**Age, Years**

Mean 63

Median 61.5

Range 49-83

**T-stage**

pT2 3 (5)

pT2a 8 (14)

pT2b 13 (23)

pT2c 13 (23)

pT3a 9 (16)

pT3b 2 (3.5)

Unknown 9 (16)

**Gleason Score**

4-6 29 (51)

7 19 (33)

8-10 8 (14)

Unknown 1 (2)

**PSA**

Median 6.8

<6.8 26 (46)

>6.8 25 (44)

Unknown 5 (9)

**PSA failure** 27 (47)

**N-stage**

N0/NX 57 (100)

M-stage

**M0/MX** 57 (100)

**Pathological diagnosis**

Adenocarcinoma 57 (100)
